# Structural and Optical Characterization of ZnS Ultrathin Films Prepared by Low-Temperature ALD from Diethylzinc and 1.5-Pentanedithiol after Various Annealing Treatments

**DOI:** 10.3390/ma12193212

**Published:** 2019-09-30

**Authors:** Maksymilian Włodarski, Urszula Chodorow, Stanisław Jóźwiak, Matti Putkonen, Tomasz Durejko, Timo Sajavaara, Małgorzata Norek

**Affiliations:** 1 Institute of Optoelectronics, Military University of Technology, Kaliskiego 2, 00-908 Warsaw, Poland; maksymilian.wlodarski@wat.edu.pl; 2 Institute of Applied Physics, Military University of Technology, Kaliskiego 2, 00-908 Warsaw, Poland; urszula.chodorow@wat.edu.pl; 3 Department of Advanced Materials and Technologies, Faculty of Advanced Technologies and Chemistry, Military University of Technology, Kaliskiego 2, 00-908 Warsaw, Poland; stanislaw.jozwiak@wat.edu.pl; 4 VTT Technical Research Centre of Finland, Biologinkuja 5, Espoo, P.O. Box 1000, FI-02044 VTT, Espoo, Finland; 5 Department of Physics, University of Jyväskylä, P.O. Box 35, FI-40014, Jyväskylä, Finland, timo.sajavaara@jyu.fi

**Keywords:** atomic layer deposition (ALD), ZnS thin films, annealing, optical properties

## Abstract

The structural and optical evolution of the ZnS thin films prepared by atomic layer deposition (ALD) from the diethylzinc (DEZ) and 1,5-pentanedithiol (PDT) as zinc and sulfur precursors was studied. A deposited ZnS layer (of about 60 nm) is amorphous, with a significant S excess. After annealing, the stoichiometry improved for annealing temperatures ≥400 °C and annealing time ≥2 h, and 1:1 stoichiometry was obtained when annealed at 500 °C for 4 h. ZnS crystallized into small crystallites (1–7 nm) with cubic sphalerite structure, which remained stable under the applied annealing conditions. The size of the crystallites (*D*) tended to decrease with annealing temperature, in agreement with the EDS data (decreased content of both S and Zn with annealing temperature); the *D* for samples annealed at 600 °C (for the time ≤2 h) was always the smallest. Both reflectivity and ellipsometric spectra showed characteristics typical for quantum confinement (distinct dips/peaks in UV spectral region). It can thus be concluded that the amorphous ZnS layer obtained at a relatively low temperature (150 °C) from organic S precursor transformed into the layers built of small ZnS nanocrystals of cubic structure after annealing at a temperature range of 300–600 °C under Ar atmosphere.

## 1. Introduction

Zinc sulfide (ZnS) is one of the most important semiconductor, widely used in optoelectronics [1], lasers and photovoltaics [2], and photocatalysis [3]. The broad interest in ZnS has its origin in its properties. Among those properties, the wide and direct band gap (3.6–3.8 eV), high refractive index at room temperature (2.35), and high transmittance from visible to infrared (up to 12 μm) range, are the most attractive features [4,5]. Because of its high dielectric constant, excellent insulation properties and exceptional chemical stabilities, ZnS is considered as one of the most important passivation materials for semiconductor devices, especially for high-aspect ratio arrays based on HgCdTe [6,7]. Recently, ZnS quantum dots (QDs) have attracted considerable attention because of their broad biomedical (fluorescent probes) and sensor applications [8,9,10,11,12].

Future devices will require a technique with a perfect conformability to prepare ZnS layers on any complex surface. Atomic layer deposition (ALD) is a technique which is characterized by conformability incomparable to other techniques. Thanks to the self-limiting reaction at the surface between gaseous precursor molecules and chemical groups at a substrate [13,14], uniform layers can be grown on high aspect ratio and three-dimensionally-structured materials. ZnS was one of the earliest ALD-grown materials [15]. For preparing ZnS films by ALD, various zinc (Zn)-precursors are used, including ZnCl_2_ [16,17], ZnMe_2_ [18,19], and ZnEt_2_ [20,21]. Among the Zn-precursors, diethylzinc (ZnEt_2_ or DEZ) is particularly attractive, owing to its high vapor pressure and possibility to grow ZnS even at low deposition temperatures [22,23,24]. Unlike the wide range of accessible Zn-precursors, the S-precursor for ZnS is normally a H_2_S compound [25], which is a very flammable and highly toxic gas. The toxicity and high tendency for contamination during deposition are strongly unwelcome, particularly for biomedical and sensing applications. Therefore, a safer S-precursor is especially needed. Recently, a new organic precursor, 1,5-pentanedithiol (PDT) for sulfur, was proposed by Ko et al. [26]. In their work, ZnS films were successfully grown on substrates with an atomic precision by repeating self-limiting chemisorption of DEZ and PDT as precursors of Zn and S, respectively. The obtained ZnS layers were, however, amorphous. Since most ZnS applications require crystalline or polycrystalline forms of ZnS (better for optical or opto-electrical performance), here, we studied the structural and optical evolution of the ZnS thin films prepared from DEZ and PDT after various annealing treatments. The structural changes of the ZnS films grown from the DEZ and PDT compounds have not been studied before. Moreover, ALD-deposited ZnS films are known to exist in both cubic (sphalerite) and hexagonal (wurtzite) crystal forms. It has been shown that the ZnS films deposited at temperatures lower 400 °C are mainly cubic, whereas the ones deposited above 400 °C are hexagonal [20,27]. The cubic and hexagonal phase can also coexist in a wide temperature range (225–400 °C) [24]. The aim of this study was also to check whether the ZnS layer undergoes a phase transformation from cubic to hexagonal crystallographic form under certain annealing conditions.

## 2. Materials and Methods 

The ZnS thin films were deposited on silicon (Si) substrate by ALD technique using a Picosun SUNALE R-200 ALD reactor in a single wafer mode. Depositions were carried out at 150 °C with diethylzinc (DEZ) and 1,5-pentanedithiol (PDT) as precursors. PDT was evaporated from a Picohot 200 hot source held at 55 °C. DEZ was kept at 20 °C to obtain surface-sufficient vapor pressure. Nitrogen (99.999%) was used as a carrier and purging gas. Typical ALD deposition cycle consisted of DEZ pulse/purge/PDT pulse/purge with 0.2s/4s/0.3s/4s timing, respectively. The ZnS film deposition rate was 0.09 Å/cycle when measured after 500–2500 deposition cycles, resulting in film thickness of ca. 60 nm.

The samples were annealed in a R80/750/12-B170 Nabertherm tube furnace under argon (99.999%) atmosphere. Five samples were prepared and investigated: one sample without annealing (“as-deposited” sample) and four samples subjected to annealing at temperatures of 300, 400, 500, and 600 °C, with increasing annealing time ranging between 0.5–4 h.

Microanalysis of chemical composition was done using a field-emission scanning electron microscope FE-SEM (FEI, Quanta) equipped with an energy-dispersive X-ray spectrometer (EDS). The chemical composition analysis was performed at 5 kV, magnification of 200, spot of 2.0, and with a constant distance of samples to the detector (WD = 10). Each measurement was repeated three times and an average of the three measurements was taken to determine the chemical composition of the studied samples.

The composition of selected as-deposited thin films was determined by means of time-of-flight-elastic recoil detection analysis (TOF-ERDA) [28,29]. In this method, a heavy 11.9 MeV^63^Cu^6+^ ion beam is directed to the sample and the time of flight (velocity) and energy of recoiled sample atoms are measured with a detector telescope at 41° to the beam direction. Independent measurement of the velocity and energy of recoil atoms allows them to be differentiated according to their mass. The elemental depth profiles were determined by means of known geometry, elastic scattering cross-sections, and stopping forces [30].

X-ray diffraction (XRD) analysis was performed using the grazing incidence X-ray diffraction (GI XRD) technique based on the Co K_α1_ radiation (λ= 1.78892 Å) using a Rigaku Ultima IV system with parallel beam mode of radiation. The crystal size and strain was calculated based on the Halder–Wagner theory (1) which, in contrast to the Williamson–Hall theory, offers more reliable data for reflections at low and intermediates angles than those at higher diffraction angles [31]:

(1) $${\left(\frac{\beta }{\tan\theta }\right)}^{2}=\frac{K*\lambda }{D}*\;\frac{\beta }{tan\theta *sin\theta }+16\varepsilon^{2}$$

where:

β—integral breadth, depending on crystallite size and microstrain (in radians);
*ϴ*—Bragg angle (in radians);
K—shape factor (dimensionless);
λ—wavelength of the X-ray (in Angstrom);
D—crystallite size (in Angstrom);
ε—microstrain ($\varepsilon =\frac{\Delta d}{d},\;\mathrm{where}\;d\;\mathrm{is}\;\mathrm{lattice}\;\mathrm{plane}\;\mathrm{spacing})$.

The ellipsometry measurements were carried out using a Sentech SE 850 variable angle spectroscopic ellipsometer. The data were acquired at an angle of incidence of 70°. The system is mainly used for thin-film characterization over a broad spectral range of 240–2400 nm. As a result of ellipsometric measurements, two parameters Ψ and Δ are obtained. Parameter Ψ is the amplitude ratio of p- and s-polarizations, and Δ is the phase difference between p- and s-polarizations. The Ψ and Δ parameters are defined from the ratio of the amplitude reflection (r_p_,r_s_) or transmission (t_p_, t_s_) coefficients for p- and s-polarizations [32]: $\frac{r_{p}}{r_{s}}=\tan\left(\Psi \right)\exp\left(i\Delta \right)$ or $\frac{t_{p}}{t_{s}}=\tan\left(\Psi \right)\exp\left(i\Delta \right)$.

Roughness of sample surfaces was determined using an optical profiler (Veeco / Wyko NT1100). For measurements, a phase shifting interferometry (PSI) mode was utilized because the thickness of thin films was ca. 60 nm, thus we expected roughness to not be higher than a few nanometers. PSI mode is used to measure topographies of smooth surfaces (discontinuities on the surface have to be less than 150 nm). The vertical resolution of measurements in PSI mode can achieve sub-nanometer scale. In this mode, a monochromatic light is used, and the surface topography is obtained by measuring the position and shape of the interference fringes on the sample.

The reflectance spectra of the samples annealed for 0.5 h were measured using a PerkinElmer Lambda 900 spectrometer with a PELA 1001 integrating sphere. Theses samples were measured in the 250–800 nm range. Other samples were measured using a PerkinElmer Lambda 650 spectrometer with a 150 mm integrating sphere in the 200–800 nm range with 1 nm step and resolution and 500 ms integration time. A beam condenser was used because of small dimensions of samples. The sphere was configured to collect both specular and diffused reflectance. Clean Si sample was used as 100% reflection reference.

## 3. Results and Discussion

**Figure 1:**
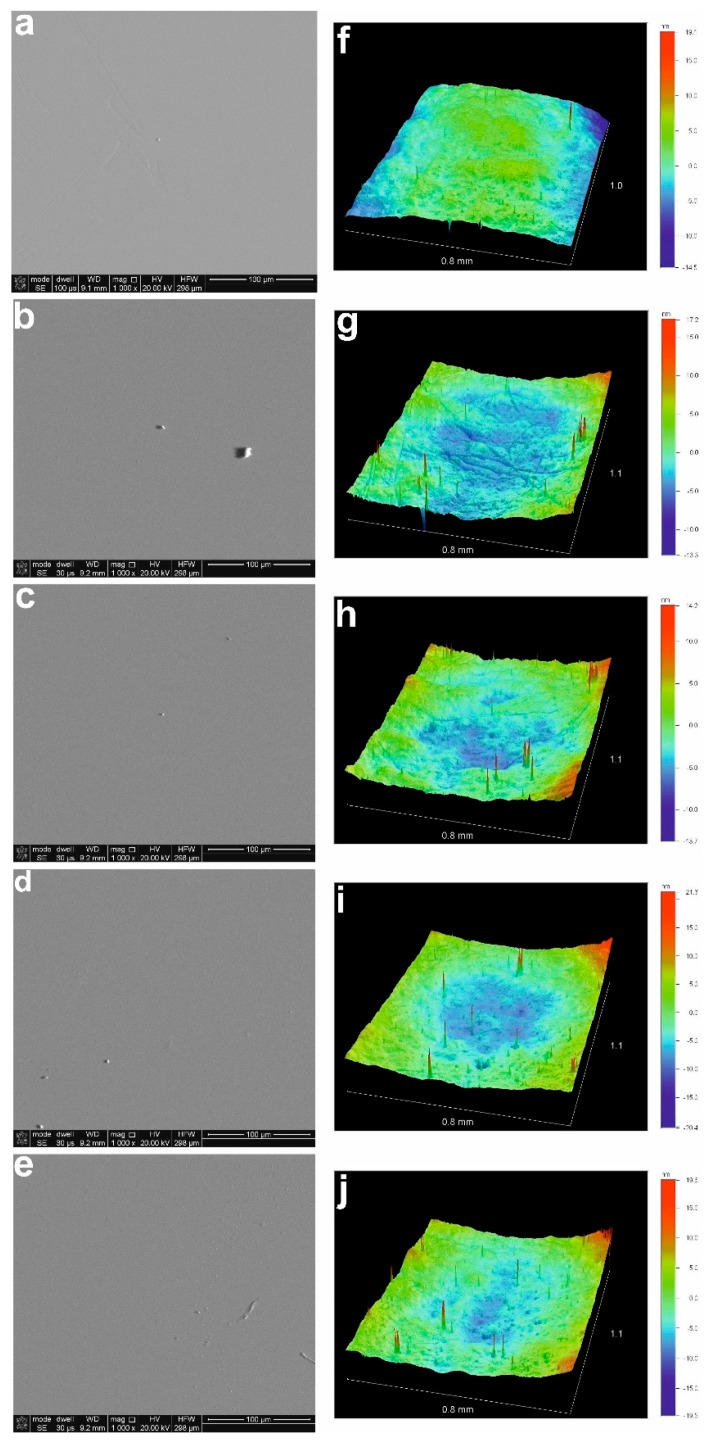
SEM images (left column) and 3D outputs obtained with Veeco/Wyko NT-1100 TM real-time optical surface profiler (right column) for ZnS film before annealing (**a**,**f**), and after annealing for 1 h at 300 °C (**b**,**g**), 400 °C (**c**,**h**), 500 °C (**d**,**i**), and 600 °C (**e**,**j**), respectively.

In Figure 1, SEM images (left column) and 3D outputs obtained with a Veeco/Wyko NT-1100 TM real-time optical surface profiler (right column) for ZnS film before annealing (Figure 1a,f), and after annealing for 1 h at 300 °C (Figure 1b,g), 400 °C (Figure 1c,h), 500 °C (Figure 1d,i), and 600 °C (Figure 1e,j) respectively, are demonstrated. As can be seen, the surface of ZnS films is very smooth, without any visible cracks or defects, before as well as after annealing treatment. 

**Figure 2:**
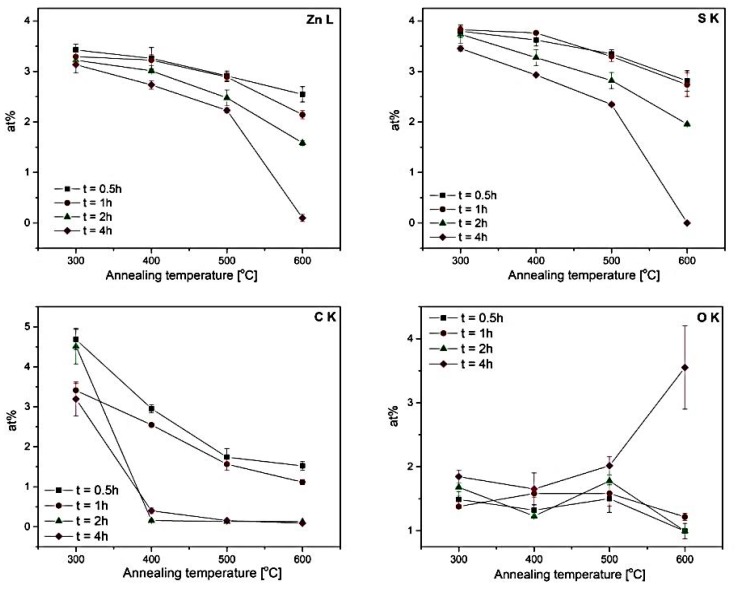
Energy-dispersive X-ray spectrometer (EDS) elemental analysis at accelerating voltage of 5 kV: the average Zn, S, C, and O concentration (at%) in the ZnS layers after annealing for 0.5, 1, 2, and 4 h as a function of annealing temperature.

Figure 2 shows the average content of zinc (Zn), sulfur (S), carbon (C), and oxygen (O) measured by EDS as a function of annealing temperature for various annealing times. The capital Roman letters K and L stand for the characteristic X-ray lines resulting from electron transitions between inner orbits in a given element. According to TOF-ERDA measurements, as-deposited films had a 1:2 Zn:S ratio and relatively high C and H content of 51 an 27 at%, respectively. This indicates relative low reactivity of the precursors, as depositions at 350 °C resulted in 1:1 stoichiometric films with H and carbon content < 1.5 and < 0.5 at%, respectively. Oxygen content was 3 at% when deposited at 150 °C and decreased to 1.5 at% at 350 °C. However, a clear decrease of the Zn, S, and C concentration of films deposited at 150 °C during annealing was observed. During annealing, a drop of C content indicated the removal of remaining impurities from precursors with uncomplete reactions. The content of carbon dropped to below the detection limit after annealing at 400, 500, and 600 °C for 2 and 4 h. The samples annealed for shorter times (0.5 and 1 h) and at relatively low temperatures (300 °C) nonetheless demonstrated a relatively large amount of carbon, suggesting that the annealing conditions were not sufficient to get rid of the residual carbon. Oxygen remained more-or-less at the similar level for all samples, except for the one with no presence of Zn, S, and C elements, which was most probably linked with an oxygenation of the Si substrates after exposure to air. 

In Table 1, the Zn and S contents in all samples are compared. First of all, the content of both Zn and S dropped significantly in the samples subjected to annealing procedures when compared to the sample without annealing (“as deposited” sample). Moreover, the decrease of S was definitely more pronounced (more than 2.5 times) compared with the decrease of Zn, which may be associated with the lower vapor pressure of the former. Nevertheless, in most of the samples, an excess of S over Zn can be observed. The stoichiometry improved for annealing temperatures ≥400 °C and annealing time ≥2 h, and it was almost perfect for the sample annealed at 500 °C for 4 h. The excess of S over Zn in the samples annealed at shorter times (0.5 and 1 h), accompanied by the relatively large amount of C in these samples, meaning that the precipitation of semiconductor nanocrystallites most probably occurred in a matrix of unreacted 1,5-pentanedithiol used as precursors of S. 

**Figure 3:**
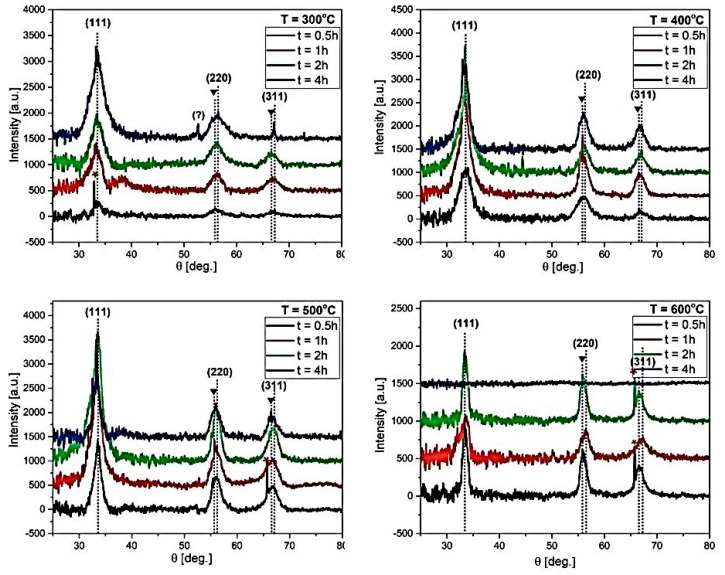
X-ray diffraction (XRD) pattern of the samples annealed at temperatures 300, 400, 500, and 600 °C for various annealing times (t) of 0.5, 1, 2, and 4 h. The red star symbol signifies the reflections originating from the Si substrate; the inverted triangles indicate the probable reflections from the twinned sphalerite.

Figure 3 shows XRD patterns of the samples annealed at temperatures (*T*) of 300, 400, 500, and 600° as a function of annealing time. The sample before annealing (the “as-deposited” sample) was amorphous (no reflection in the XRD pattern was detected). The positions of the most pronounced peaks in the patterns corresponded to the (111), (220), and (311) crystallographic planes of the cubic ZnS (PDF number 01-072-4841). In some samples, the reflection from the Si substrate was also visible (marked by a red star, PDF Number 00-027-1402). The position of the (220) and (311) peaks varied in about 0.6 degrees, which can be ascribed to the presence of twinned sphalerites in some of the samples, or the fact that ZnS crystallites are built of a mixture of both sphalerites and densely twinned sphalerites [33,34]. It was suggested that the formation of the (111)-twinned sphalerite would occur at a high temperature and with a rapid crystal-growth rate [34]. Heating of the thin ZnS films after ALD deposition could favor the rapid crystal growth rate, and thus the conditions to form the twins. The peak designated by the question mark in brackets (the sample annealed at 300 °C for 4 h) could not be identified and was ascribed to neither of the possible crystallographic phases. It could, therefore, have come from an accidental reflection from the edge of the sample or from the sample holder. Moreover, from the XRD patterns, it can be inferred that the crystallinity of the samples improved systematically with annealing time and temperature (the XRD peak intensity increased). In general, the diffraction peak intensity was determined by the arrangement of atoms in the entire crystal [35,36]. Therefore, the sharper and more intense peaks meant a better ordering of atoms in the ZnS cubic cell. For the sample annealed at 600 °C for 4 h, there was no XRD reflections, owing to the evaporation of the Zn and S elements under the prolonged annealing time at this high temperature (Figure 2). 

**Figure 4:**
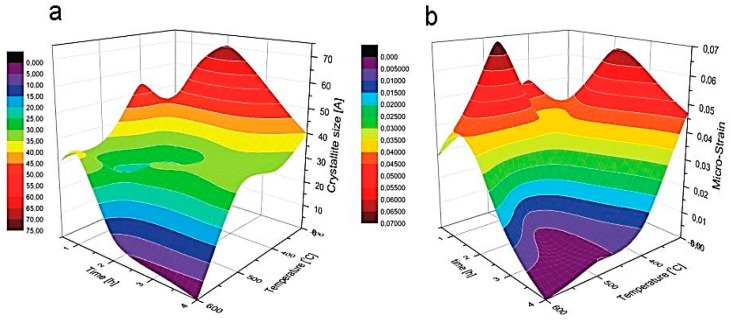
3D graphs representing **c**rystallite size (**a**) and micro-strain (**b**) as a function of annealing time and temperature for the studied ZnS thin films.

In Table 2, apart from the peak position and broadening, the crystallite size (*D*) and micro-strain values, calculated using the Halder–Wagner method, are gathered. The data are also illustrated in Figure 4. It can be seen that for the annealing time ≥2 h, there was a tendency to decrease the crystallite size and lattice strain with annealing temperature. This tendency was, however, not so obvious for the samples annealed for shorter times (0.5 and 1 h), although the *D* for samples annealed at 600 °C (for the time ≤2 h) was always the smallest. This may have been due to the presence of a large amount of carbon and the excess of sulfur in those samples (Figure 2), which could favor a formation different from cubic solid state phases. Owing to a strong repulsion between S and C [37], and negligible mutual solubilities of Zn, S [38], and C [39], a formation of graphite, monoclinic β-sulfur or hexagonal close-packed (hcp) zinc was more probable in this configuration. A small portion of these crystallographic forms, although not detectable by XRD, could influence the peak shape from the cubic (111), (220), and (311) crystallographic planes and make it difficult to determine both crystallite size and lattice strain parameters.

**Figure 5:**
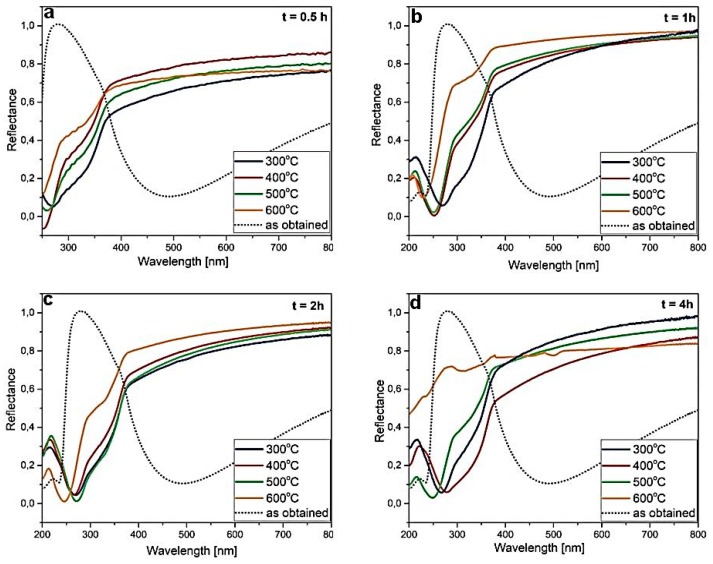
Reflectance spectra of the ZnS thin films before annealing (as obtained), and annealed at 300 °C, 400 °C, 500 °C, and 600 °C for 0.5 (**a**), 1 (**b**), 2 (**c**), and 4 h (**d**).

Figure 5 illustrates the reflectance spectra of the ZnS thin films before and after annealing treatments. As can be seen, the reflectance spectrum of the amorphous, un-annealed ZnS layer was completely different than that for the annealed ZnS thin films. The reflectance spectrum of the amorphous ZnS thin film demonstrated a broad and distinct dip in the 450–500 nm range with a reflectance value of about 0.1. Similar reflectance minimums were observed for the ZnS/Au/Ti multilayer structure deposited on the Si substrate [40] and in electrodeposited ZnS thin films after annealing treatments [41]. Moreover, a significant transmittance of ~70% at around 540 nm [42] and ~90% at around 550 nm [43] was observed for the ZnS layer thickness of 64 and 40 nm, respectively. The broad minimum at around 500 nm in the reflectance spectra of un-annealed ZnS was most probably related with the absorption of the light by Zn^2+^ ions [44,45,46,47]. After annealing of the samples, the reflectance dip shifted to the UV region and became more narrow, with the reflectance values close to zero. This was certainly associated with the crystallization of the ZnS layers upon annealing, as observed by XRD measurements (Figure 3). Similar spectra were observed for the ZnS quantum dots (QDs) of the size ranging between ~2 and 5 nm [48,49,50,51,52,53]. Therefore, the presence of distinct dips in the UV spectral region strongly suggests quantum confinement of ZnS semiconductor. Upon annealing, the ZnS layer seemingly crystallizes into small nanocrystals. 

Moreover, sharp peaks appearing below 300 nm indicated a presence of reasonably monodispersed particle size distribution. It can be also observed that for a given annealing time, the minimum had a tendency to shift towards lower wavelengths with increasing annealing temperature —it was always placed in the range of 250–300 nm for the annealing temperatures of 300–500 °C, whereas it is shifted beneath 250 nm for the samples annealed at 600 °C (except for the sample annealed at 600 °C for 4 h). In QDs, the band-gap between the valence state and the empty state increases as the nanocrystal becomes smaller [54]. Therefore, the shift of the peak in the reflectance spectra to the UV region indicates the decrease of the crystallite size and the increase of the band-gap. Although the general tendency of decreasing the crystallite size with annealing temperature as determined from the XRD data agreed well with the observed blue shift of the dip in the reflectance spectra, it is difficult to directly compare the change of crystal size from Table 2 with the position of the dips in Figure 5. This is because of a possible formation of SiOx layer of variable thickness on Si substrates that can affect the optical spectra. Furthermore, it has to be remembered that the Halder–Wagner model [31] is based on X-ray diffraction line broadening and provides more approximation than actual crystallite size values.

**Figure 6:**
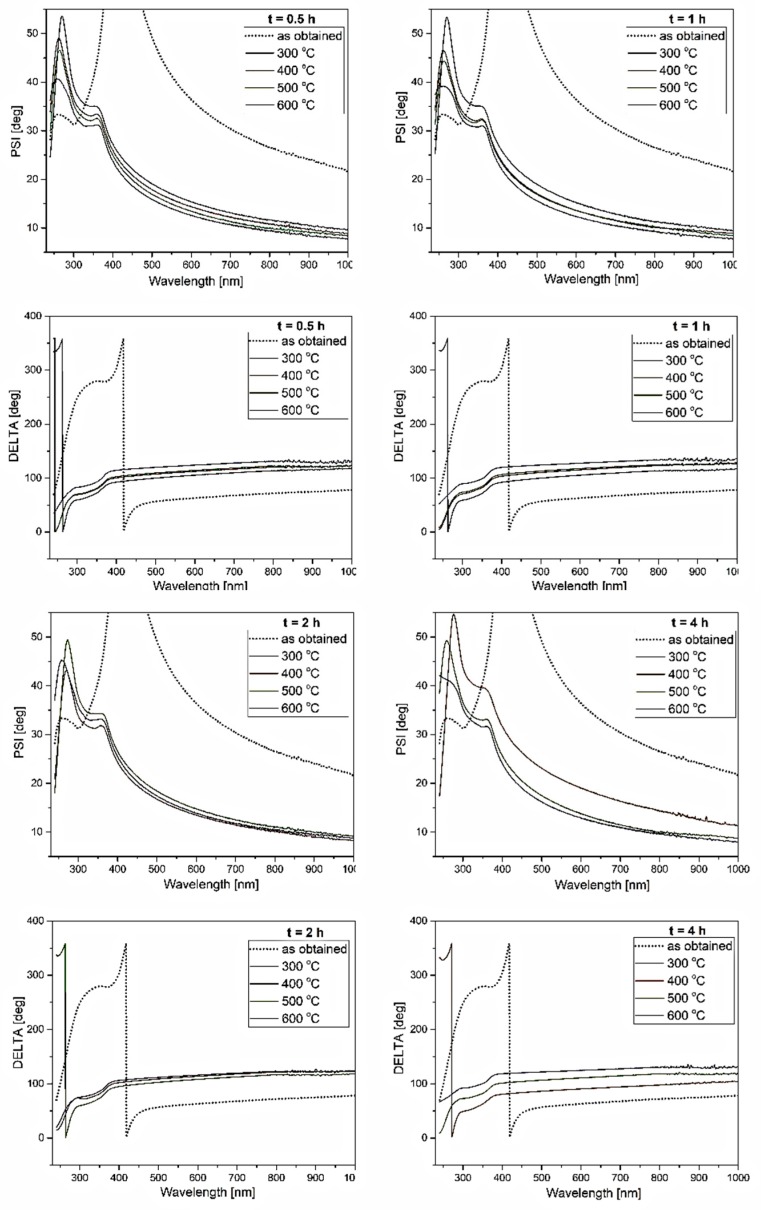
Spectral ellipsometric data (Ψ, ∆) of ZnS thin films before and after various annealing procedures.

**Figure 7:**
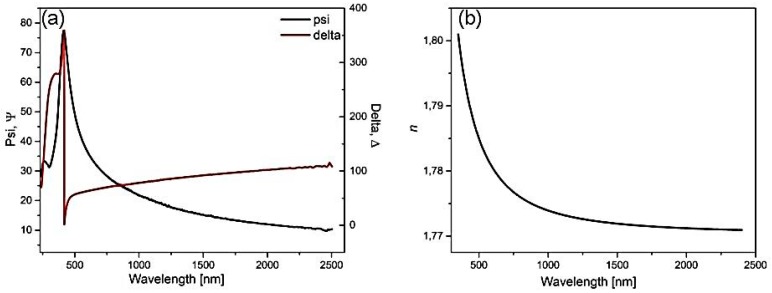
Spectral ellipsometric data (Ψ, ∆) of ZnS thin film before annealing (**a**) and refractive index (*n*) extracted from the spectral dependencies of the (Ψ, ∆) (**b**).

In Figure 6 and Figure 7, spectroscopic ellipsometry data (Ψ, ∆) of ZnS films before and after annealing are shown. Ellipsometry measures the change in polarized light upon light reflection on a sample (or light transmission by a sample). When light scattering by surface roughness reduces the reflected light intensity severely, ellipsometry measurement becomes difficult, as ellipsometry determines a polarization state from its light intensity. Therefore, if the size of the surface roughness is more than ~30% of a measurement wavelength, the measurement error increases [55]. The RMS (root mean square) surface roughness of ZnS layers was in the range of 2–5 nm (Table 3); therefore, much below the 30% mark. It can thus be concluded that the surface roughness was not a restriction in the studied cases. 

In general, the interpretation of measurement results is rather difficult from the absolute values of (Ψ, ∆). Thus, construction of an optical model is required for data analysis. The spectral dependencies of (Ψ, ∆) of amorphous ZnS layer were successfully fitted with the Sellmeier dispersion model to extract the layer thickness and refractive index (*n*) (Figure 5). The Sellmeier model is described by Equation (2) [56]:

(2) $$n^{2}\left(\lambda \right)=1+\frac{A\lambda^{2}}{\lambda^{2}-B^{2}},\;k=0$$

where λ is the wavelength, A and B are fitting parameters, and *k* is the extinction coefficient. 

The ellipsometric analysis was done by taking into account a three-layer model: upper (rough) layer (modeled with effective medium approximation, EMA), middle - ZnS layer (Sellmeier model), and silicon substrate (fixed *n*). On the basis of the analysis, the best fit was obtained for A = 2.134 and B = 0.006, and the layer thickness of the ZnS was 60.9 ± 0.5 nm (in agreement with the number of ALD cycles). The calculated refractive index of ZnS film was *n* = 1.7794 ± 0.0031 for λ = 632.8 nm. The determined refractive index was lower than that for a bulk ZnS (*n* = 2.3605 for the λ = 632.8 nm), which can be attributed to lower packing density of the amorphous thin ZnS film [57,58].

In Figure 6, the spectral ellipsometric data of the annealed ZnS thin films are compared with the (Ψ, ∆) of ZnS thin films before annealing. From the measured (Ψ, ∆) spectra it can be inferred that the optical features of ZnS layers changed significantly after annealing. In particular, the amplitude of the Ψ oscillation (peak) decreased and shifted together with ∆ to the UV spectral region. It was shown previously that both parameters (Ψ, ∆) shifted towards a shorter wavelength as the refractive index (*n*) and film thickness (*t*) decreased; however, as compared to Ψ, the ∆ was more sensitive to the changes of both *n* and *t* [59]. The decrease of ZnS layer thickness after annealing would agree with EDS analysis (evaporation of residual precursor and/or other elements, Figure 2). Thickness-dependent interference between multiple reflections from different interfaces was not visible in these cases, because of the small film thickness. Moreover, in the high-energy region, optical interference was negligible because light absorption in samples generally increased and penetration depth of light became smaller. From the analysis of this energy region, band structure and effect of surface roughness can be studied [55]. This region was very similar to the reflectivity spectra presented in Figure 7. Particularly, the peak in Ψ(λ) spectra behaved exactly the same as the dip in the reflectivity spectra—it tended to shift towards shorter wavelengths with annealing temperature, suggesting a decrease in the particle size (along with the layer thickness decrease) and the increase of the bandgap. Therefore, the ellipsometric data corroborated the assumption that a ZnS layer after ALD deposition at a relatively low temperature crystallizes into small ZnS nanocrystals of cubic structure after annealing at temperatures in the range of 300–600 °C, with optical characteristics typical for quantum confinement.

**Figure 8:**
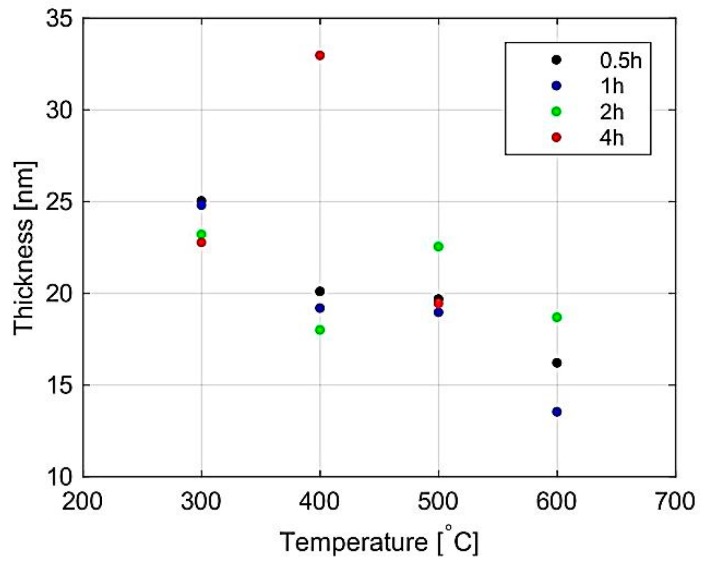
Thickness of the ZnS films as a function of annealing temperature for a given annealing time extracted from the spectral dependencies of the (Ψ, ∆).

The ellipsometric data of the annealed samples could not be fitted with the three-layer model; thus, other models were tested [60,61,62,63]. The best fit was obtained by taking into account the four-layer model: upper - rough layer (modeled with effective medium approximation, EMA), ZnS layer (Cody–Lorenz function), SiOx layer (Sellmeier model), and silicon substrate (fixed *n*). Both Cody–Lorenz (CL) as well as Tauc–Lorenz (TL) models were applied for amorphous and polycrystalline semiconductor thin films, having strong interband transitions; however, the CL is considered to be more accurate because of an Urbach absorption tail and modified density of states [64,65]. In this work, the CD model was found to yield better values of optical constants and lower mean square error (MSE), as compared to the TL model. The resulted parameters are gathered in Table S1 (Supplementary Materials). As expected, thickness (*t*) of the ZnS layer dropped significantly after annealing—for a given annealing time, *t* tended to decrease with the annealing temperature (Figure 8). 

**Figure 9:**
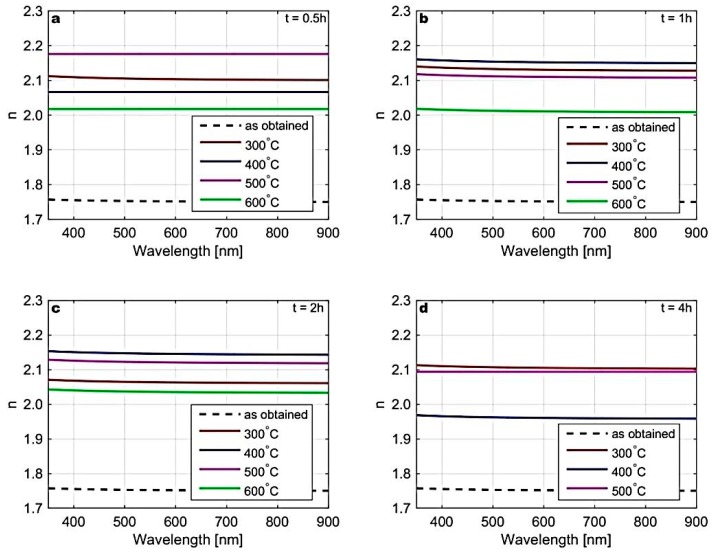
Refractive index (*n*) extracted from the spectral dependencies of the (Ψ, ∆) measured for the ZnS thin films before annealing (as obtained), and annealed at 300 °C, 400 °C, 500 °C, and 600 °C for 0.5 (**a**), 1 (**b**), 2 (**c**), and 4 h (**d**).

In Figure 9, the spectral dependencies of refractive index (*n*) extracted from the spectral dependencies of the (Ψ, ∆) presented in Figure 6 are provided (except for the sample annealed at 600 °C for 4 h where the amount of Zn and S elements were close to zero, as determined by the EDS analysis). It can be seen that there was a significant increase of *n* after annealing. In some cases, *n* became close to that of the bulk ZnS. However, *n* did not show any strict dependence on the applied annealing parameters, although *n* for the sample annealed at 600 °C was always the smallest. This was probably caused by a complexity and inhomogeneity of the films (the presence of ZnS nanocrystals embedded in a mixture of various phases) that could not be included in this simplistic four-layer model.

## 4. Conclusions

ALD of ZnS layer from the diethylzinc and 1,5-pentanedithiol at 150 °C resulted in growth of an amorphous layer with a significant excess of S over Zn element. After annealing, the S excess in films was maintained; however, the stoichiometry improved for annealing temperatures ≥400 °C and annealing time ≥ 2 h, and it was almost stoichiometric for the films annealed at 500 °C for 4 h. For annealing at 600 °C for 4 h, the Zn and S elements completely evaporated. ZnS crystallized into small crystallites (1–7 nm) with cubic sphalerite structure. The cubic phase remained stable—ZnS film did not undergo a phase transformation from cubic to hexagonal form under the applied annealing conditions. The size of the crystallites (*D*) tended to decrease with annealing temperature in agreement with the EDS data (decreased content of both S and Zn with annealing temperature)—the *D* for samples annealed at 600 °C (for the time ≤ 2 h) was always the smallest. The reflectivity spectra of the ZnS thin films after annealing were characterized by distinct dips in the UV region. The position of the dip tended to shift towards shorter wavelengths, with the annealing temperature most probably owing to the decrease of the ZnS crystallite size and, consequently, a wider band-gap (for the samples annealed at 600 °C the dip was shifted below 250 nm). Similar results were obtained from ellipsometric measurements, where the shift of the peak in Ψ(λ), together with the shift of ∆ to the UV spectral region, was observed for the annealed samples. The shift towards a shorter wavelength occurred when the refractive index (*n*) and film thickness (*t*) decreased. The thickness of the ZnS amorphous layer before annealing treatment was determined using the Sellmeier model and was around 60 nm. The reduction of the film thickness implied a reduction in the size of the ZnS crystallites. Therefore, it can be concluded that the amorphous ZnS layer obtained at relatively low temperature (150 °C) from organic S precursor transformed into the layers built of small ZnS nanocystals after annealing at a temperature range of 300–600 °C under Ar atmosphere, with optical characteristics typical for quantum confinement. 

## Figures and Tables

**Table 1:** The average Zn and S concentration (at%) in Zn–S thin films measured by EDS at 5 kV.

Temp	Zn L					S K				
	As Obtained	0.5 h	1 h	2 h	4 h	As Obtained	0.5 h	1 h	2 h	4 h
−	3.65 ± 0.04					8.75 ± 0.23				
300 °C	−	3.42 ± 0.11	3.29 ± 0.14	3.22 ± 0.06	3.14 ± 0.15	−	3.80 ± 0.12	3.83 ± 0.06	3.74 ± 0.18	3.45 ± 0.05
400 °C		3.26 ± 0.22	3.22 ± 0.11	3.01 ± 0.04	2.73 ± 0.08		3.62 ± 0.12	3.76 ± 0.02	3.27 ± 0.16	2.93 ± 0.01
500 °C		2.91 ± 0.01	2.89 ± 0.09	2.48 ± 0.15	2.23 ± 0.05		3.35 ± 0.08	3.30 ± 0.01	2.82 ± 0.16	2.35 ± 0.04
600 °C		2.55 ± 0.15	2.14 ± 0.08	1.58 ± 0.05	0.10 ± 0.07		2.81 ± 0.20	2.74 ± 0.23	1.96 ± 0.02	0.00 ± 0.00

**Table 2:** Peak position and broadening, crystallite size, and micro-strain parameters determined from the XRD measurements of ZnS thin films after the annealing processes.

**0.5 h**	**Peak Position (Broadening) [2θ]**			**Crystallite Size *D* [Å]**	**Micro-Strain *e* × 10^3^**
	**(111)**	**(220)**	**(311)**		
300 °C	33.07 (2.10)	55.84 (1.40)	67.05 (2.50)	37.42	38.97
400 °C	33.71 (3.00)	56.31 (02.44)	66.56 (1.00)	53.85	51.42
500 °C	33.65 (1.89)	55.98 (2.02)	66.69 (1.20)	33.73	36.14
600 °C	33.36 (1.76)	56.08 (1.90)	66.92 (1.62)	30.65	28.28
**1 h**	**Peak position (broadening) [2θ]**			**Crystallite size *D* [Å]**	**Micro-Strain *e* × 10^3^**
	**(111)**	**(220)**	**(311)**		
300 °C	33.50 (3.19)	56.31 (2.54)	66.84 (3.50)	56.61	47.50
400 °C	33.18 (1.99)	55.37 (1.80)	66.59 (2.01)	35.07	36.32
500 °C	33.28 (1.69)	56.00 (2.07)	66.97 (3.02)	23.93	69.42
600 °C	33.94 (2.10)	56.57 (1.89)	67.30 (2.08)	37.09	37.91
**2 h**	**Peak position (broadening) [2θ]**			**Crystallite size *D* [Å]**	**Micro-Strain *e* × 10^3^**
	**(111)**	**(220)**	**(311)**		
300 °C	33.40 (3.85)	56.19 (2.00)	66.61 (2.80)	70.52	65.10
400 °C	33.45 (1.75)	56.11 (2.12)	66.75 (2.20)	27.69	36.32
500 °C	33.51 (1.83)	56.04 (1.79)	67.02 (2.28)	30.81	12.50
600 °C	33.30 (0.60)	55.76 (0.69)	66.50 (1.00)	08.93	33.91
**4 h**	**Peak position (broadening) [2θ]**			**Crystallite size *D* [Å]**	**Micro-Strain *e* × 10^3^**
	**(111)**	**(220)**	**(311)**		
300 °C	33.27 (2.21)	55.84 (1,80)	67.05 (1.43)	40.04	46.90
400 °C	33.12 (1.83)	55.89 (2.06)	66.62 (1.97)	30.87	10.61
500 °C	33.35 (1.90)	55.82 (2.02)	66.41 (2.23)	31.71	05.00
600 °C	−	−	−	−	−

**Table 3:** RMS (root mean square) roughness (nm) for the studied ZnS thin films.

Temp	As Obtained	0.5 h	1 h	2 h	4 h
−	2.16	−	−	−	−
300 °C	−	1.56	4.77	2.59	4.30
400 °C		2.82	3.90	2.58	3.54
500 °C		2.32	3.54	2.28	2.12
600 °C		1.47	3.79	2.44	3.74

## Supplementary Materials

The following are available online at https://www.mdpi.com/1996-1944/12/19/3212/s1, Table S1: The best fit parameters extracted from the spectral dependencies of the (Ψ, ∆) presented in Figure 7 using the four-layer model.
