# Supplementary material for: Structural and Optical Characterization of ZnS Ultrathin Films Prepared by Low-Temperature ALD from Diethylzinc and 1.5-Pentanedithiol after Various Annealing Treatments
Source: Materials (Basel). 2019 Sep 30;12(19):3212. doi: 10.3390/ma12193212 (PMC6804116; doi:10.3390/ma12193212)
Supplement: Supplementary file 1 [file materials-12-03212-s001.pdf]

materials

Supplementary Materials: Structural and Optical Characterization of ZnS Ultrathin Films Prepared by Low-Temperature ALD from Diethylzinc and 1.5-Pentanedithiol after Various Annealing Treatments

Maksymilian Włodarski [1], Urszula Chodorow [2], Stanisław Jóźwiak [3], Matti Putkonen [4,†], Tomasz Durejko [3,*], Timo Sajavaara [5] and Małgorzata Norek [3]

[1] Institute of Optoelectronics, Military University of Technology, Kaliskiego 2, 00-908 Warsaw, Poland; maksymilian.wlodarski@wat.edu.pl

[2] Institute of Applied Physics, Military University of Technology, Kaliskiego 2, 00-908 Warsaw, Poland; urszula.chodorow@wat.edu.pl

[3] Department of Advanced Materials and Technologies, Faculty of Advanced Technologies and Chemistry, Military University of Technology, Kaliskiego 2, 00-908 Warsaw, Poland; stanislaw.jozwiak@wat.edu.pl (S.J.), tomasz.durejko@wat.edu.pl (T.D.), malgorzata.norek@wat.edu.pl (M.N.)

[4] VTT Technical Research Centre of Finland, Biologinkuja 5, Espoo, P.O. Box 1000, FI-02044 VTT, Espoo, Finland; matti.putkonen@helsinki.fi

[5] Department of Physics, University of Jyväskylä, P.O. Box 35, FI-40014, Jyväskylä, Finland, timo.sajavaara@jyu.fi

† Current address: Department of Chemistry, University of Helsinki, P.O. Box 55, FI-00014 Helsinki, Finland

* Correspondence: malgorzata.norek@wat.edu.pl; mnorek73@gmail.com (M.N.); tomasz.durejko@wat.edu.pl (T.D.); Tel.: +48-261-839-350 (M.N.); +48-261-837-135 (T.D.)

Table 1. Best fit parameters extracted from the spectral dependencies of the (Ψ, Δ) presented in Fig. 7 using the four-layer model.

**1**	**As obtained**	**MSE**	**0.69**	
	Layer Name	Thickness [nm]	Refr. Index [632.8 nm]	E_g [eV]
	Air	-	1.000	
	roughness	16.1	1.359	
	ZnS	62.5	1.752	4.735
	SiO_x	2.8	3.635	
	Silicon VIS+NIR	-	3.872	
2	300^0C, 0.5h	MSE	1.16	
	Layer Name	Thickness [nm]	Refr. Index [632.8 nm]	
	Air	-	1.000	
	roughness	0.0	1.647	
	ZnS	25.0	2.103	4.906
	SiO_x	1.4	3.919	
	Silicon VIS+NIR	-	3.872	
3	400^0C, 0.5h	MSE	1.37	
	Layer Name	Thickness [nm]	Refr. Index [632.8 nm]	
	Air	-	1.000	
	roughness	0.3	1.548	

	ZnS	20.1	2.067	-
	SiO_x	2.8	3.174	
	Silicon VIS+NIR	-	3.872	
4	500°C, 0.5h	MSE	1.87	
	Layer Name	Thickness [nm]	Refr. Index [632.8 nm]	
	Air	-	1.000	
	rough	0.0	1.608	
	ZnS	19.7	2.176	-
	SiO_x	2.2	3.241	
	Silicon VIS+NIR	-	3.872	
5	600°C, 0.5h	MSE	1.87	
	Layer Name	Thickness [nm]	Refr. Index [632.8 nm]	
	Air	-	1.000	
	roughness	0.00	1.522	
	ZnS	16.2	2.018	-
	SiO_x	2.0	3.333	
	Silicon VIS+NIR	-	3.872	
6	300°C, 1h	MSE	1.40	
	Layer Name	Thickness [nm]	Refr. Index [632.8 nm]	
	Air	-	1.000	
	roughness	0.0	1.583	
	ZnS	24.8	2.130	4.784
	SiO_x	1.5	3.791	
	Silicon VIS+NIR	-	3.872	
7	400°C, 1h	MSE	1.35	
	Layer Name	Thickness [nm]	Refr. Index [632.8 nm]	
	Air	-	1.000	
	roughness	0.0	1.594	
	ZnS	19.2	2.152	4.339
	SiO_x	1.9	3.014	
	Silicon VIS+NIR	-	3.872	
8	500°C, 1h	MSE	1.38	
	Layer Name	Thickness [nm]	Refr. Index [632.8 nm]	
	Air	-	1.000	
	roughness	0.0	1.571	
	ZnS	19.0	2.110	4.595

	SiO_x	1.8	3.470	
	Silicon VIS+NIR	-	3.872	
9	600^{0}C, 1h	MSE	1.81	
	Layer Name	Thickness [nm]	Refr. Index [632.8 nm]	
	Air	-	1.000	
	roughness	0.4	1.517	
	ZnS	13.5	2.010	4.759
	SiO_x	2.8	2.901	
	Silicon VIS+NIR	-	3.872	
10	300^{0}C, 2h	MSE	1.03	
	Layer Name	Thickness [nm]	Refr. Index [632.8 nm]	
	Air	-	1.000	
	roughness	0.0	1.546	
	ZnS	23.2	2.063	4.768
	SiO_x	2.6	3.373	
	Silicon VIS+NIR	-	3.872	
11	400^{0}C, 2h	MSE	1.96	
	Layer Name	Thickness [nm]	Refr. Index [632.8 nm]	
	Air	-	1.000	
	roughness	0.1	1.591	
	ZnS	18.0	2.145	
	SiO_x	4.1	3.002	4.818
	Silicon VIS+NIR	-	3.872	
12	500^{0}C, 2h	MSE	1.46	
	Layer Name	Thickness [nm]	Refr. Index [632.8 nm]	
	Air	-	1.000	
	roughness	0.0	1.577	
	ZnS	22.5	2.120	4.872
	SiO_x	2.3	3.236	
	Silicon VIS+NIR	-	3.872	
13	600^{0}C, 2h	MSE	1.73	
	Layer Name	Thickness [nm]	Refr. Index [632.8 nm]	
	Air	-	1.000	
	roughness	2.0	1.531	
	ZnS	18.7	2.035	4.187
	SiO_x	1.9	3.609	

Silicon VIS+NIR	-	3.872	
14 300^{0}C, 4h	MSE	1.35	
Layer Name	Thickness [nm]	Refr. Index [632.8 nm]	
Air	-	1.000	
rough	0.5	1.569	
ZnS	22.8	2.105	5.017
SiO_x	2.2	3.309	
Silicon VIS+NIR	-	3.872	
15 400^{0}C, 4h	MSE	1.80	
Layer Name	Thickness [nm]	Refr. Index [632.8 nm]	
Air	-	1.000	
roughness	0.5	1.490	
ZnS	33.0	1.960	4.923
SiO_x	3.0	3.439	
Silicon VIS+NIR	-	3.872	
16 500^{0}C, 4h	MSE	2. 57	
Layer Name	Thickness [nm]	Refr. Index [632.8 nm]	
Air	-	1.000	
roughness	0.2	1.563	
ZnS	19.4	2.094	-
SiO_x	4.2	3.076	
Silicon VIS+NIR	-	3.872	

The applied model overestimated *n* values for SiO_x layer: in some cases the refractive index for SiO_x layer is larger than 3. The model overestimated also a few values of the band-gap energy (Eg), therefore, these values are omitted in the Table 1S. This is probably caused by a complexity and inhomogeneity of the films (the presence of ZnS nanocrystals embedded in a mixture of various phases) that could not be included in the simplistic four-layer model.
